# A Single-Camera-Based Three-Dimensional Velocity Field Measurement Method for Granular Media in Mass Finishing with Deep Learning

**DOI:** 10.3390/s24154790

**Published:** 2024-07-24

**Authors:** Jie Zou, Chunyue Tian, Yiqun Liu, Junfei Ding, Wenhui Li

**Affiliations:** 1 College of Aeronautics and Astronautics, Taiyuan University of Technology, Jinzhong 030600, China; zoujie7837@link.tyut.edu.cn (J.Z.); 2023511333@link.tyut.edu.cn (Y.L.); wenhui_li7190@126.com (W.L.); 2 College of Mechanical and Vehicle Engineering, Taiyuan University of Technology, Taiyuan 030024, China

**Keywords:** mass finishing, granular flow, 3D velocity field measurement, LiteFlowNet, single-camera method

## Abstract

Surface treatment processes such as mass finishing play a crucial role in enhancing the quality of machined parts across industries. However, accurate measurement of the velocity field of granular media in mass finishing presents significant challenges. Existing measurement methods suffer from issues such as complex and expensive equipment, limited to single-point measurements, interference with the flow field, and lack of universality in different scenarios. This study addresses these issues by proposing a single-camera-based method with deep learning to measure the three-dimensional velocity field of granular flow. We constructed a complete measurement system and analyzed the accuracy and performance of the proposed method by comparing the measurement results with those of the traditional DIC algorithm. The results show that the proposed method is very accurate in measuring spatial displacement, with an average error of less than 0.07 mm and a calculation speed that is 1291.67% of the traditional DIC algorithm under the same conditions. Additionally, experiments in a bowl-type vibratory finishing machine demonstrate the feasibility of the proposed method in capturing the three-dimensional flow of granular media. This research not only proposed a novel method for three-dimensional reconstruction and velocity field measurement using a single-color camera, but also demonstrated a way to combine deep learning with traditional optical techniques. It is of great significance to introduce deep learning to improve traditional optical techniques and apply them to practical engineering measurements.

## 1. Introduction

Mass finishing is an important surface treatment process that involves placing components in a container filled with granular media, grinding fluid, and water. Through a certain form of relative motion, it achieves collision, rolling pressure, micro-grinding, and certain chemical actions on the surface of the workpiece. As a result, it accomplishes polishing, brightening, deburring, cleaning, and degreasing effects, thus enhancing surface quality [1]. Due to its advantages such as strong adaptability to components, good processing effects, economic efficiency, and environmentally friendly processes, mass finishing is widely used in surface treatment of machined parts in various industries [1,2].

Many studies have shown that the contact force and impact velocity between the granular media and the workpiece directly affect the processing effect in mass finishing [3,4,5,6]. However, the granular media are a collective of particulate matter influenced by contact force, damping, and gravity, and exhibit dynamic characteristics of forced particle groups. These characteristics of granular media flow result in velocity measurement methods that are different from traditional measurement methods for solid, liquid, and gas [7].

Existing velocity measurement methods for granular flow can be roughly classified into five categories.

The first category involves obtaining contact force through in-situ measurement and deriving velocity information from the contact force data. Ciampini’s team has made significant contributions in this area. They developed a method to extract normal impact velocity from measured impact force signals [3] and creatively utilized the deformation of Almen strips under impact to characterize the effect of impact velocity [8]. The methods of this category have certain limitations, such as the need for expensive high-sampling rate equipment to record contact force and the fact that the measurement area can only be a single point in the flow field.

The second category involves using various fiber-optical probes (FOPs) and laser-Doppler velocimetry (LDV) to detect the flow field velocity. For example, Hashemnia et al. [9] developed a laser displacement probe for measuring the velocity of mass-finishing flow fields, while Liu et al. [10] developed a novel optical-fiber probe for detecting local instantaneous solid-volume concentration, velocity, and flux information in the flow field. The limitations of this category include the need for complex setup, expensive equipment, and a high-performance processor for real-time signal processing. Additionally, FOPs and LDV can only measure a very small volume in the flow field.

The third category is modeling of the flow field. The most commonly used methods are discrete-element method (DEM)-based and computational fluid dynamics (CFD)-based modeling of the granular flow field. Ciampini’s team [8], Hashimoto’s team [11,12], and Uhlmann’s team [13] have conducted research in this area. The limitations of this category are very obvious. First, building a specific model is complex and difficult. Second, perfecting a model will consume a large amount of time, but a specific model cannot be used in different scenarios. Additionally, these types of models, which are based on traditional algorithms, will cause a significant computational load.

The fourth category consists of some special methods. For example, Domblesky et al. [14] passed a knotted nylon rope through a small hole in the workpiece and determined the actual velocity field of individual objects by calculating the motion time and length of the nylon rope. Hashimoto et al. [11] attached the end of a fishing line to a granule and allowed the fishing line to move with the medium. By measuring the elongation of the fishing line during motion, they obtained the average velocity of the medium’s movement. These methods interfere with the flow field and can only obtain the velocity data of a few granules. Therefore, they are only used in some specific scenarios.

The fifth category is methods based on optical techniques. The measurement of fluid velocity fields using optical techniques is very mature and has been conducted by many researchers. However, the studies on using optical techniques to measure the velocity field of clustered solid granular media flow are scarce. Some researchers have used particle-image velocimetry (PIV) and particle-tracking velocimetry (PTV) techniques from experimental fluid dynamics to measure the velocity of granular flow [15,16,17], but there are still few substantive results published in the literature. Among these few researchers, two teams have achieved substantial results. Thomas Hagemeier et al. compared the four techniques of FOPs, LDV, PIV, and PTV in the measurement of a particle flow velocity field in a fluidized bed and summarized the capabilities, limitations, and development potential of these techniques [18]. J. X. Duan et al. used a deep learning network to measure the two-dimensional velocity field of granules in rotary drums [19]. Both teams used very advanced optical methods, but their work was limited to single-point or two-dimensional measurements of the flow field.

In actual mass finishing, granular flow is a complex three-dimensional flow, and the flow parameters in different areas are significantly different. Therefore, developing a global three-dimensional velocity field measurement method for granular flow is very important for studying the processing effect of mass finishing. As there is currently no global three-dimensional velocity field measurement method for granular flow, we propose a single-camera-based three-dimensional velocity field measurement method for granular media in mass finishing with deep learning. This method uses optical flow [20,21] to process images obtained using the single-camera multi-view method proposed by Lee’s team [22] to reconstruct the three-dimensional flow and velocity field. In addition, we innovatively introduce an optical flow deep learning network [23,24] to replace the traditional optical flow algorithm, which significantly reduces the computing time and the requirements for computing hardware resources.

The method we proposed is able to analyze images based on temporal and spatial transformations to obtain the overall velocity of the granular flow. We constructed the corresponding measurement system and demonstrated the accuracy and performance of our method by comparing the results with those of the traditional DIC algorithm. Through the measurement of granular media in a bowl-type vibratory finishing machine, we proved that our method can efficiently and accurately measure the three-dimensional velocity field of granular flow in mass finishing. Furthermore, our research not only provides a novel measurement method for three-dimensional granular flow with one single-color camera, but also highlights the great research potential of integrating deep learning with traditional optical techniques.

## 2. Three-Dimensional Velocity Field Measurement System

### 2.1. Composition of the Measurement System

**Figure 1:**
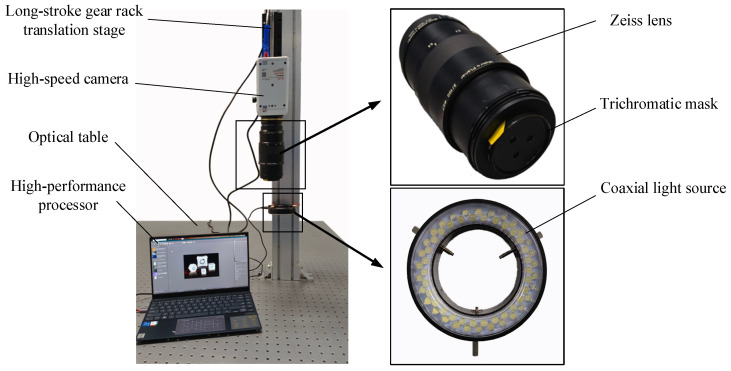
Three-dimensional velocity field measurement system.

As illustrated in Figure 1, the three-dimensional velocity field measurement system for granular media comprises two main components: hardware and software systems. The hardware system consists of a high-speed camera (the parameter settings of the high-speed camera are shown in Table 1), a lens (Zeiss Milvus 2/100M ZF.2-mount), a trichromatic mask, a coaxial light source, a long-stroke gear rack translation stage (Zolix AD160C-40), and a high-performance processor. The software system includes a proprietary algorithm platform designed specifically for measuring the three-dimensional velocity field of granular media.

**Figure 2:**
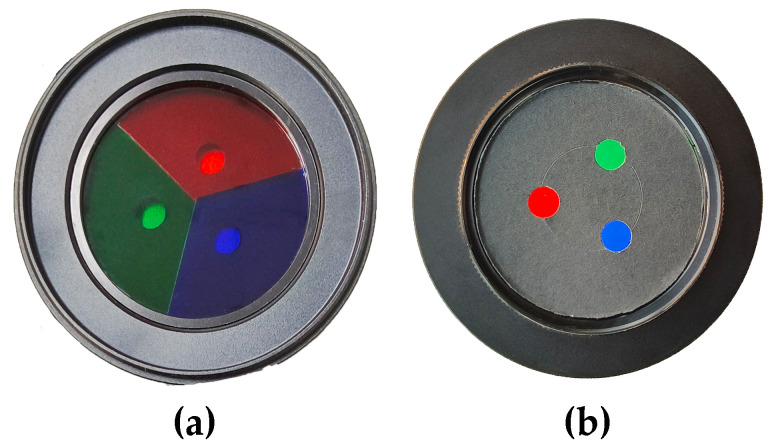
The trichromatic mask. (**a**) is the side facing into the lens, and (**b**) is the side facing out of the lens.

It is necessary to introduce the composition and function of the self-designed trichromatic mask here. The trichromatic mask consists of three 1/3 circular filters (as shown in Figure 2a) and three circular apertures (as shown in Figure 2b, the center distance between every two apertures is 19 mm). Each of the red, green, and blue 1/3 circular filters only allows light with wavelengths of 650 nm, 532 nm, and 450 nm to pass through, respectively. The trichromatic mask serves to convert the three RGB channels into three perspectives of a single camera. This is achieved by using a specific algorithm to separate the RGB channels from the captured images, resulting in images from three perspectives of a single camera. Then, the flow field is reconstructed using multi-perspective three-dimensional reconstruction technology (more details about multi-perspective three-dimensional reconstruction will be introduced in the following section). This approach can reduce hardware investment in the experimental system and eliminate the requirement for high precision synchronization when using multiple cameras [25].

### 2.2. Principle of Multi-Perspective Three-Dimensional Reconstruction

**Figure 3:**
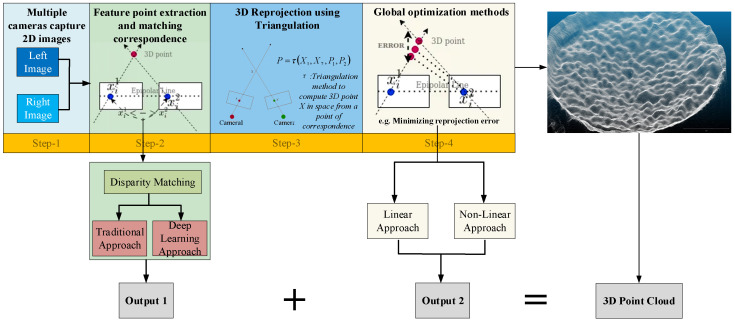
Schematic diagram of the principle of traditional MVS.

Multi-perspective three-dimensional reconstruction is an image-based technique used to reconstruct the three-dimensional shape and structure of an object from multiple two-dimensional images taken from different perspectives. This technology has been greatly developed in the past few decades, and now there are many widely used methods such as EPI [26], PMVS [27], Depth Estimation CNNs [28], etc. Figure 3 is a schematic diagram showing the principle of the traditional multi-view stereo (MVS) method, which involves the following key steps:

Firstly, multiple cameras are utilized to capture two-dimensional images of the target object from different perspectives. In this paper, multiple cameras are replaced by the aforementioned trichromatic mask and a single-color camera. These images need to be preprocessed, including image registration and noise reduction, to ensure image quality and accurate alignment. 

Secondly, feature point extraction and matching are performed. Using image processing algorithms such as SIFT or SURF, significant feature points are extracted from each image. Subsequently, matching algorithms like nearest-neighbor search are employed to identify corresponding feature point pairs across different perspective images. For this feature extracting and matching step, this paper uses an optical flow estimation convolutional neural network (CNN) named LiteFlowNet to replace the traditional method (details in Section 2.5), which not only greatly shortens the calculation speed, but also does not sacrifice the resolution of the original images.

Thirdly, triangulation is applied. Given the intrinsic and extrinsic parameters of the known camera, as well as the pixel coordinates of corresponding feature points in different images, the three-dimensional positions of these feature points can be computed. Specifically, triangulation uses geometric relationships to solve for the intersection points of rays in three-dimensional space, thereby determining the three-dimensional coordinates of the feature points. Normally, triangulation is used to recover the depth information *z* of a three-dimensional point $P\left(x,y,z\right)$ by observing the point at different positions and exploiting the triangular relationship between the two-dimensional projection points $X_{1}\left(x_{1},y_{1}\right)$ and $X_{2}\left(x_{2},y_{2}\right)$ observed at different positions. For example, suppose there are two cameras with projection matrices $P_{1}$ and $P_{2}$ and feature points in the images with coordinates $X_{1}\left(x_{1},y_{1}\right)$ and $X_{2}\left(x_{2},y_{2}\right)$, respectively. The coordinates of the three-dimensional point $P\left(x,y,z\right)$ can be determined using the following equations:

(1) $$X_{1}=P_{1}P$$

(2) $$X_{2}=P_{2}P$$

(3) $$P=\tau (X_{1},X_{2},P_{1},P_{2})$$

where τ denotes the triangulation method [29].

Finally, global optimization methods such as bundle adjustment (BA) are used to further refine the reconstructed three-dimensional point cloud by minimizing the reprojection error as determined by the following equation [30]:

(4) $$\underset{\left\{P_{i},P_{j}\right\}}{min}\;\sum_{i=1}^{n}\sum_{j=1}^{m}\rho \left(∥X_{ij}-P_{j}P_{i}{∥}^{2}\right).$$

where $P_{i}$ represents the three-dimensional coordinates of the i-th point, $P_{j}$ represents the projection matrix of the j-th camera, $X_{ij}$ represents the observed two-dimensional projection point of the i-th three-dimensional point in the j-th camera image, *n* is the total number of three-dimensional points, *m* is the total number of cameras, and ρ repressents the loss function. This step significantly enhances the accuracy and consistency of the reconstruction results.

Through these steps, multi-perspective three-dimensional reconstruction generates a three-dimensional point cloud, which can be used for further three-dimensional model reconstruction, measurement, and analysis.

### 2.3. Principle of Operation for the Measurement System

The operation principle of the hardware system is illustrated in Figure 1. The assembled camera is mounted on the long-stroke gear rack translation stage, powered on, and connected to the high-performance processor. The long-stroke gear rack translation stage and the camera lens are adjusted to ensure that the camera’s capture area covers the desired range. The image capture interval ∆*T* is set in the camera control software and the camera is used to capture images of the granular flow field in the measurement area.

**Figure 4:**
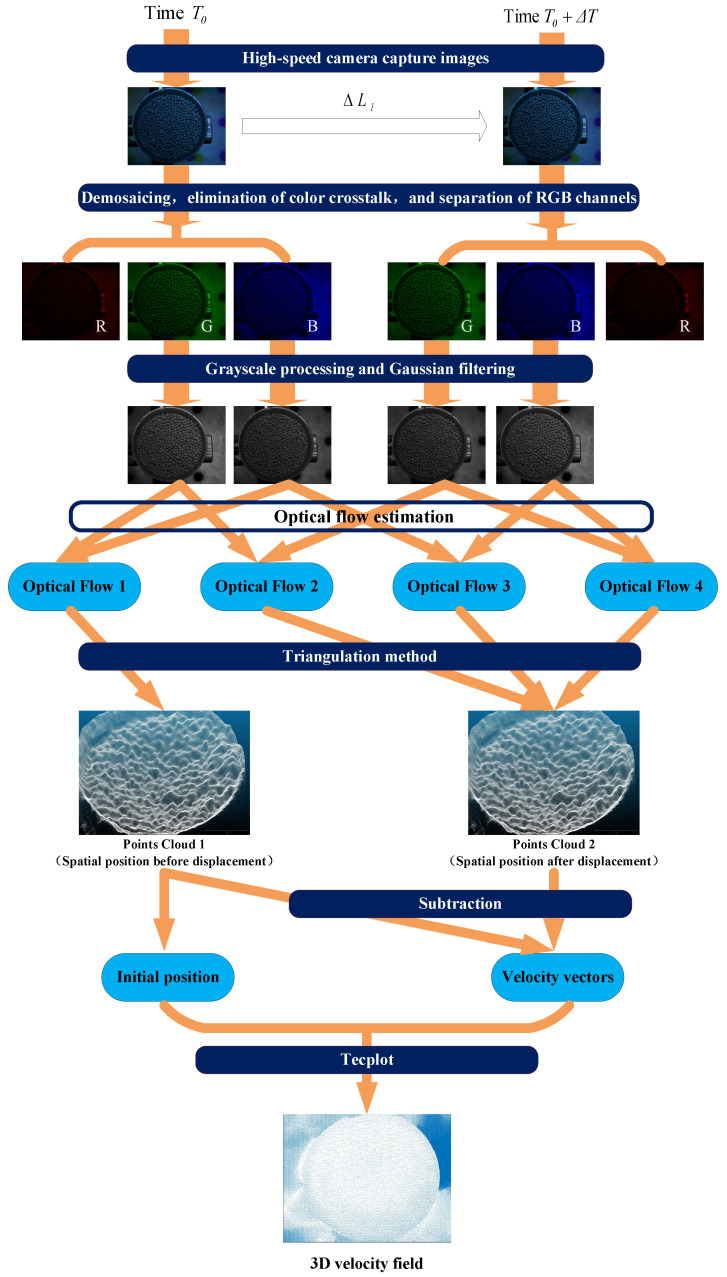
The operation principle of the software system.

The operational principle of the software system is shown in Figure 4, which relies on a self-designed algorithm platform for measuring the three-dimensional velocity field of the granular media. Camera calibration is performed using pre-captured images of a checkerboard calibration board to obtain the camera’s intrinsic and extrinsic parameters. Based on this algorithm, the three-dimensional positions $L_{n}$ and $L_{n+1}$ of the granular flow field in space at time instants $T_{n}$ and $T_{n+1}$ are calculated. Using the positions of the granular flow field at different time instants, the displacement ∆*L* in space is calculated, and then, the three-dimensional velocity *V* of the granular flow field is determined. The formula for calculating the three-dimensional velocity of the granular flow field is as follows:

(5) $$V=\frac{∆L}{∆T}$$

(6) $$∆T=T_{n+1}-T_{n}$$

(7) $$∆L=L_{n+1}-L_{n}.$$

Finally, visualization processing is performed on the spatial positions of the granular flow field, as well as the three-dimensional velocity field of the granular media.

### 2.4. Algorithm Workflow of the Measurement System

**Figure 5:**
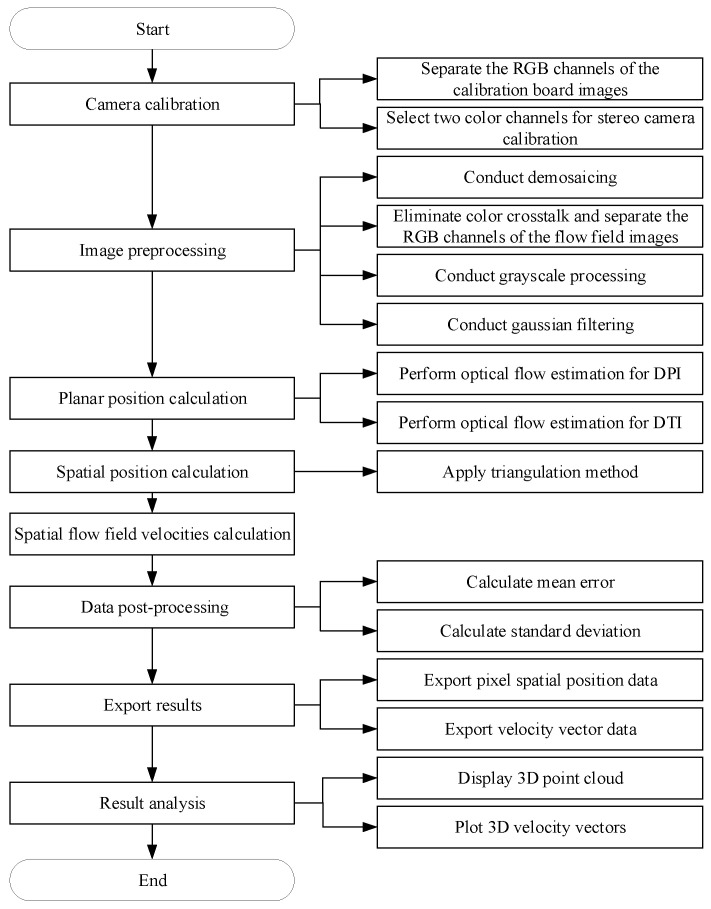
Algorithm Flowchart.

By summarizing and supplementing the operation principle of the software system shown in Figure 4, the algorithm flow of the measurement system can be obtained as shown in Figure 5. Prior to obtaining motion images of the granular flow field, parameters of the camera’s spatial position and viewpoint need to be acquired for application of the subsequent triangulation algorithm. Calibration is conducted using the Stereo Camera Calibrator toolbox in MATLAB (version: R2021a). During calibration with the Stereo Camera Calibrator toolbox, the RGB channels of the checkerboard calibration board image are first separated using an algorithm to obtain images from the three perspectives. Subsequently, calibration is performed using two of the three perspectives (the specific details will be presented in Section 3.2). 

Prior to calculating the planar position, several factors affecting the calculation results need to be addressed by preprocessing the images. This includes the following steps:

- Demosaicing helps restore the details and clarity of the image, reducing errors caused by capturing moving objects [31,32].
- Eliminating color crosstalk is performed to more effectively restore the true colors of the image, ensuring color accuracy and quality. The process of eliminating color crosstalk also involves separating the RGB channels of the motion images of the granular flow field [33,34].
- Grayscale processing is employed to address the issue of slow computation speed due to the large amount of data involved in the calculation of the granular media three-dimensional velocity field.
- Gaussian filtering is applied to remove noise introduced during the image acquisition process by the imaging equipment, transmission devices, and shooting environment.

The planar position calculation is a crucial step in the granular media three-dimensional velocity field measurement algorithm, which employs an optical flow estimation convolutional neural network (CNN)—LiteFlowNet.

To differentiate between different steps of the planar position calculation there are two distinct estimations performed. Different perspectives images (DPI) optical flow estimation is performed on images separated into different perspectives and captured by the camera at a specific moment. Different time images (DTI) optical flow estimation is performed on images separated into the same perspective and captured by the camera at different moments.

After obtaining the results of the planar position calculation, the three-dimensional position of the granular media in space is computed using the triangulation method combined with camera calibration parameters.

A target area containing granular media is selected for measured displacement averaging, and the mean errors of the measurement results are obtained by subtracting the actual applied displacement values. This step aims to evaluate the accuracy of the measurement results. Additionally, standard deviation (SD) detection is applied to the measurement results. Standard deviation (SD) is a measure of the spread of the data distribution, quantifying the extent to which the data values deviate from the arithmetic mean. A smaller standard deviation (SD) indicates less deviation of the data values from the mean.

Finally, the data results are exported, and CloudCompare (version: v2.12.4) and Tecplot 360 (version: 2022 R1) software are employed for visualizing the granular flow field, facilitating a more intuitive presentation of the research findings.

### 2.5. LiteFlowNet

LiteFlowNet is a lightweight optical flow estimation neural network model in deep learning, which provides efficient and accurate optical flow estimation. “Optical flow” is a technique in computer vision for computing pixel motion in a scene. It calculates the motion information of each pixel point in the scene by analyzing the correlation between pixels in two consecutive images [19,20]. The model is designed with lightweight and practicality in mind, enabling it to operate in resource-constrained environments.

**Figure 6:**
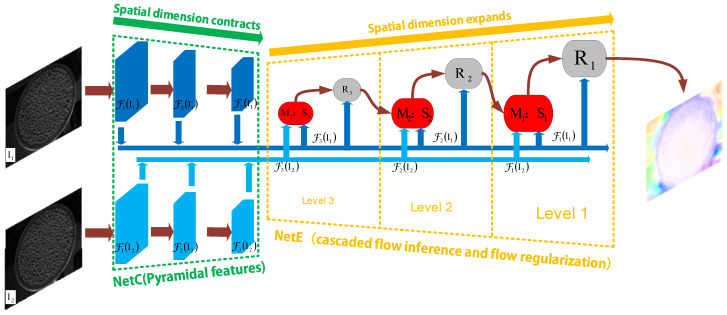
The network structure of LiteFlowNet. Both NetC and NetE are designed with six levels. For the purpose of illustration, only a three-level design is shown in this figure. When given an image pair ($I_{1}$ and $I_{2}$), NetC generates two pyramids of high-level features ($F_{i}\left(I_{1}\right)$ in dark blue and $F_{i}\left(I_{2}\right)$ in light blue). Then, NetE generates multi-scale flow fields through a cascaded flow inference module M:S (in red, consisting of a descriptor matching unit M and a sub-pixel refinement unit S) and a regularization module R (in gray).

The LiteFlowNet used in this paper is an alternative network proposed by Tak-Wai Hui et al. [35], which serves as a replacement for FlowNet2. The structure of this network is shown in Figure 6.

LiteFlowNet consists of two compact subnetworks: the encoder network (NetC) and the decoder network (NetE). NetC is used to extract pyramid features of images, converting the given image pairs into pyramids of two multi-scale high-dimensional features. NetE consists of cascaded flow inference and regularization modules, used for estimating coarse-to-fine optical flow fields. Below are the main steps for optical flow estimation implemented in LiteFlowNet.

#### 2.5.1. Pyramid Feature Extraction

Pyramid feature extraction is a multi-scale feature extraction method used to capture image information at different scales in image processing and computer vision tasks. This approach is based on the concept of pyramids, where images are constructed into a series of images according to different resolution levels.

In pyramid feature extraction, Gaussian pyramids are commonly used to generate multi-scale representations of images. Gaussian pyramids are a series of images obtained by repeatedly smoothing and downsampling the original image, with each image layer having a lower resolution than the previous one. This multi-scale representation allows the system to analyze images at different scales, thereby improving the detection and recognition capabilities of objects or features at different scales.

Once the Gaussian pyramid is constructed, feature extraction algorithms can be applied at each scale. For example, local feature descriptors can be used at each scale to detect and describe feature points or regions in the image. These features can then be used for tasks such as object detection, object tracking, image registration, etc.

The NetC of LiteFlowNet has two streams, each acting as a feature descriptor, transforming images ($I_{1}$ and $I_{2}$) into pyramids of multi-scale high-dimensional features {$F_{i}\left(I_{1}\right)$} and {$F_{i}\left(I_{2}\right)$}. The pyramid features in NetC are generated by stride-s convolutions with the reduction of spatial resolution by a factor of *s* on the pyramid.

#### 2.5.2. Feature Warping

Feature warping is a method of feature transformation, and in the LiteFlowNet model, feature warping aims to reduce the feature space distance, thereby improving the accuracy and stability of optical flow estimation.

Specifically, by warping the high-level feature $F_{2}$ towards the low-level feature $F_{1}$ via feature warping (f-warp), the feature-space distance between $F_{1}$ and $F_{2}$ is reduced. The formula for this feature warping can be expressed as follows:

(8) $${\tilde{F}}_{2}\left(X\right)≜F_{2}\left(X+\dot{X}\right)\sim F_{1}\left(X\right),$$

where $\dot{X}$ represents the optical flow estimation. In general, for any sub-pixel displacement $\dot{X}$, $F_{2}$ is warped towards $F_{1}$ by f-warp, and the formula is as follows:

(9) $$\overset{ˇ}{F}\left(X\right)=\sum_{X_{s}^{i}\in N\left(X_{s}\right)}F\left(X_{s}^{i}\right)\left(1-\left|x_{s}-x_{s}^{i}\right|\right)\left(1-\left|y_{s}-y_{s}^{i}\right|\right),$$

where $X_{s}=X+\dot{X}={\left(x_{s}+y_{s}\right)}^{T}$ denotes the source coordinates in the input feature map F that defines the sample point, $X={\left(x,y\right)}^{T}$ denotes the target coordinates of the regular grid in the interpolated feature map $\overset{ˇ}{F}$, and $N\left(X_{s}\right)$ denotes the four pixel neighbors of $X_{s}$.

Different from traditional f-warp, the f-warp of LiteFlowNet is performed on high-level CNN features rather than directly on images, making LiteFlowNet faster and more efficient in solving optical flow problems.

F-warp allows the LiteFlowNet model to infer residual flow between $F_{1}$ and ${\overset{ˇ}{F}}_{2}$ with smaller flow magnitude, which enables accurate inference of the complete flow field. The f-warp operation is applied to each layer of the pyramid feature extraction and to each module (M:S) of cascaded flow inference, effectively improving the accuracy and stability of optical flow estimation.

#### 2.5.3. Cascaded Flow Inference

Cascaded flow inference consists of the first flow inference (descriptor matching) and the second flow inference (sub-pixel refinement).

The first flow inference is conducted within the descriptor matching unit M, where the correlation between high-level feature vectors in individual pyramid features $F_{1}$ and $F_{2}$ is computed to establish point correspondences between images $I_{1}$ and $I_{2}$. This is represented as follows:

(10) $$c\left(X,\;d\right)=F_{1}\left(X\right)\cdot F_{2}\left(X+d\right)/N,$$

where *c* is the matching cost between points *X* in $F_{1}$ and points *X* + *d* in $F_{2}$, dϵZ is the displacement vector starting from point *X*, and *N* is the length of the feature vector. The cost *C* is the sum of all matching costs. Then, the residual flow $∆{\dot{X}}_{m}$ is inferred by filtering the cost *C*, and the complete flow field ${\dot{X}}_{m}$ is computed as follows:

(11) $${\dot{X}}_{m}=\underset{∆{\dot{X}}_{m}}{\underbrace{M\left(C\left(F_{1},\;{\overset{ˇ}{F}}_{2};d\right)\right)}}+s{\dot{X}}^{↑s},$$

where, ↑s represents upsampling in spatial resolution, and *s* is a scalar magnification factor. In the first flow inference, NetE reduces computational burden and improves network speed by performing short-range matching, f-warp, and matching at sampled positions only in pyramid levels of high spatial resolution.

Following the descriptor matching, the second flow inference is introduced to refine the pixel-level flow estimation ${\dot{X}}_{m}$ from the descriptor matching unit M to sub-pixel accuracy. After warping $F_{2}$ to ${\overset{ˇ}{F}}_{2}$ by f-warp, the sub-pixel refinement unit S minimizes the feature-space distance between $F_{1}$ and ${\overset{ˇ}{F}}_{2}$ by computing the residual flow $∆{\dot{X}}_{s}$, resulting in a more accurate flow field ${\dot{X}}_{s}$ as follows:

(12) $${\dot{X}}_{s}=\underset{∆{\dot{X}}_{s}}{\underbrace{S\left(F_{1},{\overset{ˇ}{F}}_{2},{\dot{X}}_{m}\right)}}+{\dot{X}}_{m}.$$

#### 2.5.4. Flow Regularization

In machine learning, regularization is primarily used to control the complexity of a model and prevent overfitting. Deep learning models typically have a large number of parameters, and when the model becomes too complex, it may perform well on the training data but poorly on the testing data, resulting in overfitting.

The flow regularization in LiteFlowNet is achieved through a feature-driven local convolution (f-lcon) layer at each pyramid level. This layer helps to smooth the flow field and maintain clear flow boundaries by using adaptive kernels based on pyramidal features, flow estimates, and occlusion probability maps. The f-lcon filters are specialized to handle smooth variations in flow vectors while avoiding oversmoothing across flow boundaries.

## 3. Measurement Accuracy Analysis

### 3.1. Displacement Measurement System

**Figure 7:**
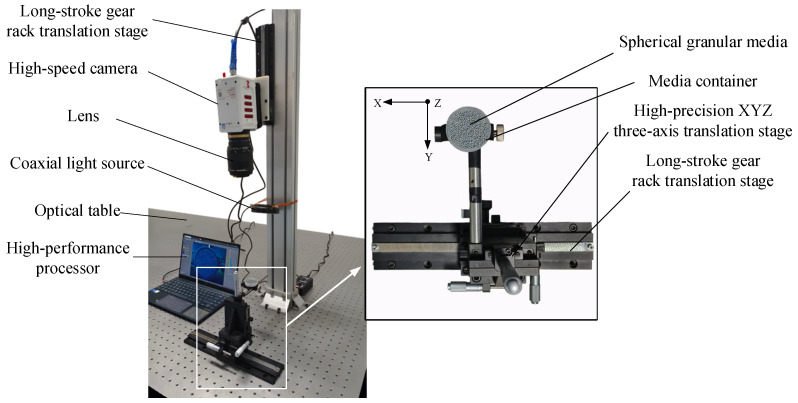
Displacement measurement system.

**Figure 8:**
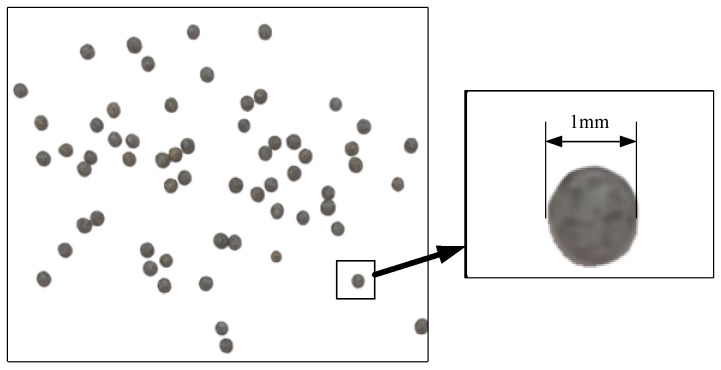
Spherical granular media.

As shown in Figure 7, the displacement measurement system has been modified from the existing measurement system by incorporating a high-precision XYZ three-axis translation stage, spherical granular media (as shown in Figure 8), and a container for the granular media.

### 3.2. Displacement Measurement Process

**Figure 9:**
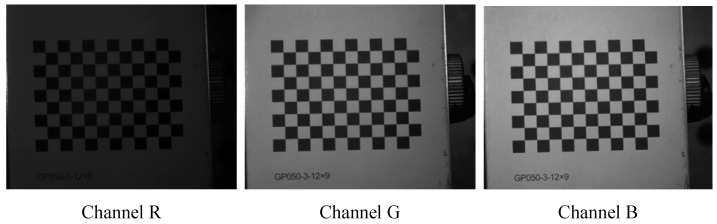
The three perspectives of a checkerboard pattern image.

Before starting the displacement measurement, adjust the camera parameters and lens to ensure clear capture of both the granular media and the checkerboard pattern. Utilize the Stereo Camera Calibrator toolbox in MATLAB to calibrate the camera, obtaining its intrinsic and extrinsic parameters as well as its relative spatial position. Figure 9 shows the three perspectives obtained by separating the RGB channels of a checkerboard pattern image. As a warm-colored light source is used in this measurement, the R-channel perspective appears relatively dark overall, hence the G-channel and B-channel should be selected for the measurement.

After completing the above preparations, start the displacement measurement process. Place the granular media inside the container and position it on the high-precision XYZ three-axis translation stage with an accuracy of 0.01 mm. The measurement is performed in two phases: first, apply displacement only in the X-axis direction, and second, apply displacement only in the Z-axis direction. Since the use of optical flow estimation network requires consecutive images or sufficiently small motion between two images, the value of pixel displacement between two images must be controlled within the computable range of the model when measuring the actual flow field motion. Based on the optical flow estimation of consecutive-displacement images, the results indicate that LiteFlowNet can compute pixel displacements ranging from sub-pixel levels to approximately 70 pixels. Considering the actual displacement value represented by a single pixel, capture one picture for every 0.5 mm displacement applied. Each set of images should total only 5 mm of displacement, resulting in two sets of images to be captured. Subsequently, preprocess these images in MATLAB. First, perform demosaicing, eliminate color crosstalk, and separate the RGB channels of the images. Then, select the G-channel and B-channel for subsequent steps, and apply grayscale conversion and Gaussian filtering to the images.

Use LiteFlowNet to estimate the optical flow between images of different perspectives and different times to obtain the planar displacements of pixels caused by target movement. Then, apply triangulation method to compute the pixel positions in space, and further calculate the three-dimensional displacements.

**Figure 10:**
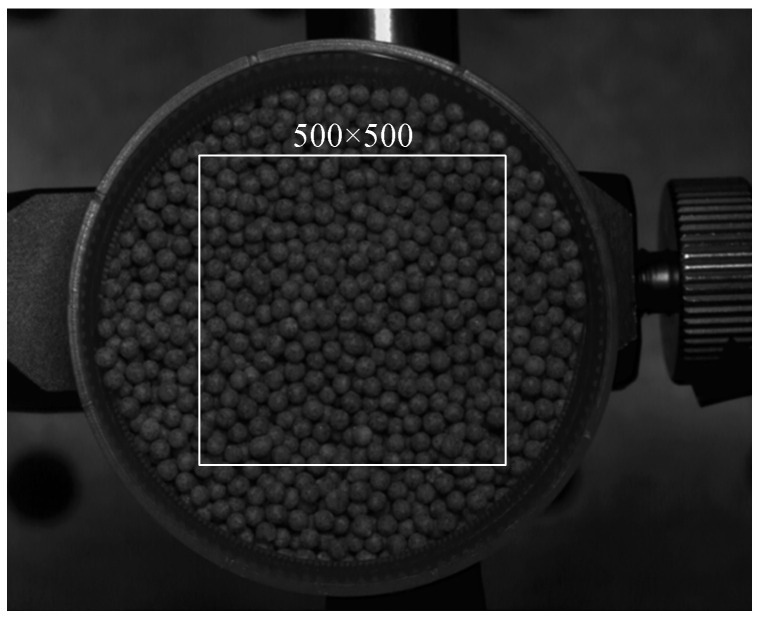
A 500×500 pixel area.

To analyze the accuracy of the measurement, select a 500×500 pixel area containing the granular media from the images (as shown in Figure 10). Average the displacements of all pixels in this area are and compare them with the actual displacement. Throughout the measurement process, no external loads or artificial disturbances should be applied to the granular media. Thus, the displacement measured for each translation should be kept consistent. 

This analysis experiment was set up to validate the accuracy and stability of the system during the translation processes.

### 3.3. Result Analysis

**Figure 11:**
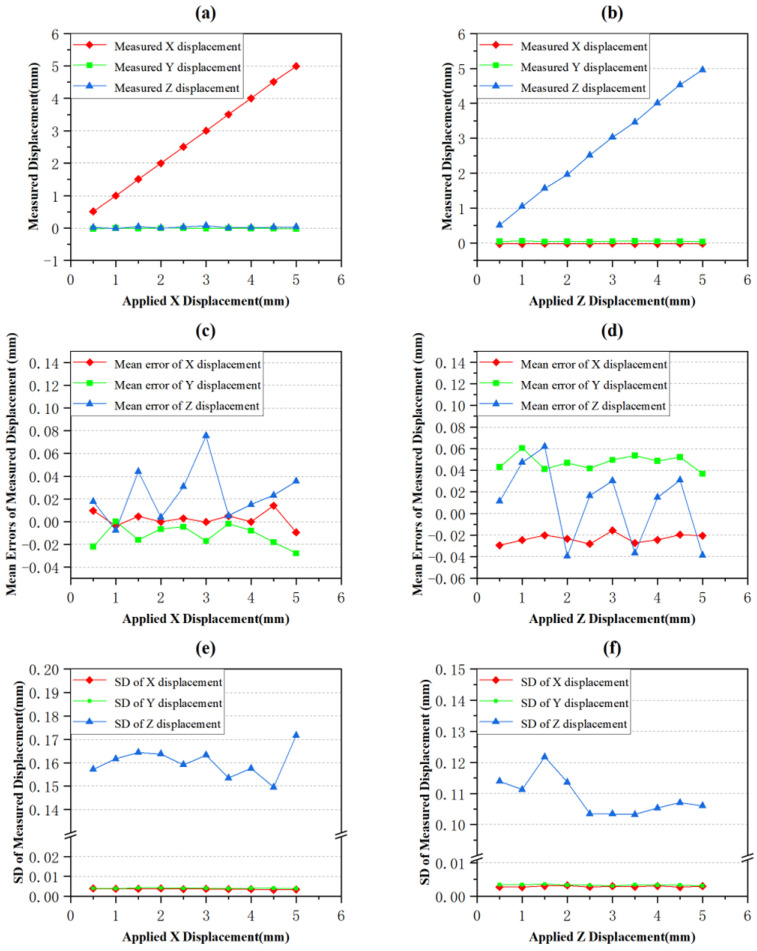
Measurement result using LiteFlowNet. (**a**,**b**) are the line graphs of measured displacement in X and Z directions, respectively. (**c**,**d**) are the line graphs of the mean errors of measured displacement in X and Z directions, respectively. (**e**,**f**) are the line graphs of the SD of measured displacement in X and Z directions, respectively.

It can be observed from Figure 11a,b that the measured displacement values in the X and Z directions match exactly with the actual applied displacement values, while the displacements in the other two directions are close to zero, indicating no applied displacement in those directions.

To assess the accuracy of the measurement, the mean error for each displacement was calculated by subtracting the applied displacement values from the measured displacement values. The resulting mean errors were then plotted in Figure 11c,d. It can be seen that the mean errors of the X direction displacement were all less than 0.02 mm, and the mean errors of the Z direction displacement were all less than 0.07 mm. In Figure 11e,f, the standard deviation (SD) of pixel displacement values in each direction within the selected 500×500 pixel area is presented. It can be observed that the standard deviations (SDs) of the displacements in the Z direction are significantly larger than these in the X and Y directions in both sets of measurements. These differences in standard deviations (SDs) are not due to random errors but are highly correlated with the hardware settings and structural parameters of the measurement system. Firstly, since the observation points in this measurement are located above the target points, larger Z-direction errors may be introduced by the triangulation method. Secondly, issues related to lighting conditions caused by the use of a warm-colored light source in this measurement, and potential optical distortions, lens distortions, sensor noise, etc. in the measurement process of the camera sensor, could contribute to the larger standard deviations (SDs) in the Z direction.

Taking into account the minor systematic errors generated during the installation of the displacement measurement system and the random errors encountered during measurement process, these errors may lead to imperfect translation of the granular media in the X and Z directions. Considering all these factors, we can conclude that the established measurement system is capable of accurately measuring the spatial displacement of the granular flow field.

## 4. Performance Analysis

In order to analyze the performance of LiteFlowNet, this section compares the results of the digital image correlation (DIC) algorithm with the results in Section 3.3.

### 4.1. Result of DIC Algorithm

**Figure 12:**
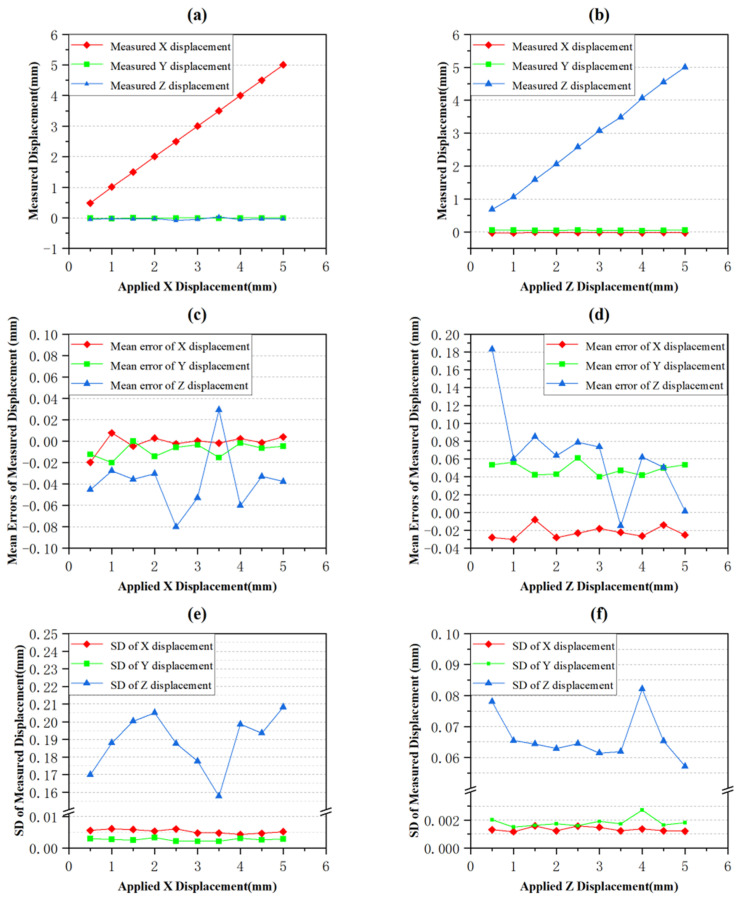
Measurement result using DIC algorithm. (**a**,**b**) are the line graphs of measured displacement in X and Z directions, respectively. (**c**,**d**) are the line graphs of the mean errors of measured displacement in X and Z directions, respectively. (**e**,**f**) are the line graphs of the SD of measured displacement in X and Z directions, respectively.

The function of DIC algorithm is the same as LiteFlowNet, which is to calculate the planar position of pixels [36]. The DIC algorithm is used instead of LiteFlowNet to process the obtained images in Section 3.2, and the results are shown in Figure 12.

### 4.2. Performance Comparison

In order to compare the accuracy of the results obtained by the DIC algorithm with the results obtained by LiteFlowNet, the important parameters shown Figure 11 and Figure 12 are summarized in Table 2.

By comparing and analyzing the error percentage data in the table, it can be observed that the measured displacement errors in the X direction are all less than 0.5%, indicating that both methods were highly accurate in measuring X-direction displacements. However, in the Z direction, the average measured displacement error using LiteFlowNet was 1.96%, while the average measured displacement error using DIC algorithm was 12.87%, showing a significant difference in accuracy between the two methods. In terms of the average value, the error when using LiteFlowNet was only 15.23% of that when using the DIC algorithm, representing an accuracy improvement of 656.63%. The main reason for this accuracy difference is that the DIC algorithm has a high dependence on the texture and contrast of the images during measurement [37]. DIC algorithm determines the pixel displacement by comparing the grayscale value distribution of image subsets. However, when a small displacement is applied to the object in the Z direction, the texture between the images is almost unchanged and the contrast is very low, which results in almost unchanged grayscale value distribution. Therefore, it is difficult for DIC algorithm to match the feature points accurately, resulting in large measurement errors. In addition, due to the large number of steps and large amount of calculation in DIC algorithm, the matching errors may be amplified, further affecting its accuracy in the Z direction. In contrast, LiteFlowNet, as a deep learning model, is able to automatically learn complex features of images during training and maintain high accuracy in many cases.

Then, comparing the SD data in the table reveals that the SDs of the measured displacements obtained by both methods are very close and far less than 1, indicating very small data fluctuations.

Next, the computation time consumption of the two methods was compared. On the same hardware device (CPU: AMD R7-5800H, GPU: NVIDIA GeForce RTX 3060 6G), the average time consumption of the LiteFlowNet and DIC algorithm to calculate the planar displacements of 1,310,720 pixels in a pair of 1024×1280 images were 1.2 s and 15.5 s, respectively. Thus, the calculation speed of LiteFlowNet was 1291.67% of that of DIC algorithm. Since the computation time consumption of the DIC algorithm depends on the step size and subset size, the result of 15.5 s was obtained by using the step size and subset size while ensuring the same output accuracy as LiteFlowNet.

## 5. Experimental Application

### 5.1. Experiment Setup

**Figure 13:**
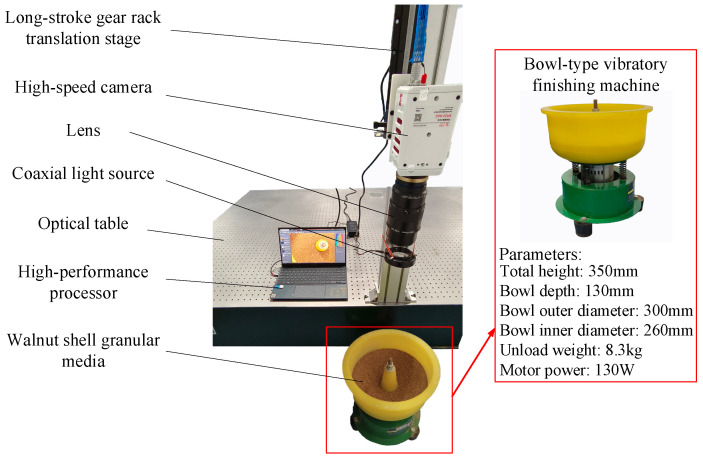
Three-dimensional velocity field measurement experiment setup.

As shown in Figure 13, the experiment setup has been modified from the existing measurement system by incorporating a walnut shell granular media and a bowl-type vibratory finishing machine.

**Figure 14:**
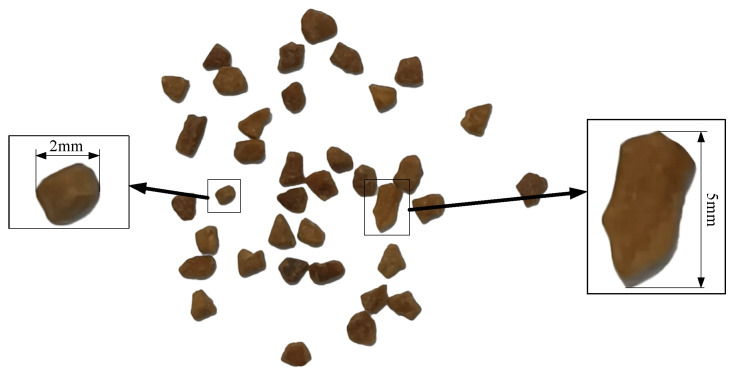
Walnut shell granular media.

Figure 14 shows the details of the walnut shell granular media. The shapes of the granules are irregular, with diameters ranging from 2 to 5 mm.

### 5.2. Experiment Process

Before the experiment starts, adjust the camera parameters to ensure the clarity of the shooting area of the granular flow field. When the experiment starts, first start the bowl-type vibratory finishing machine, and wait for the machine to run until the motion of the granular media becomes stable before proceeding with the subsequent steps. Use the camera control software to control the camera to capture images of the flow field in motion. Then, process the obtained images to obtain the three-dimensional velocity field of the granular media.

### 5.3. Experiment Result

**Figure 15:**
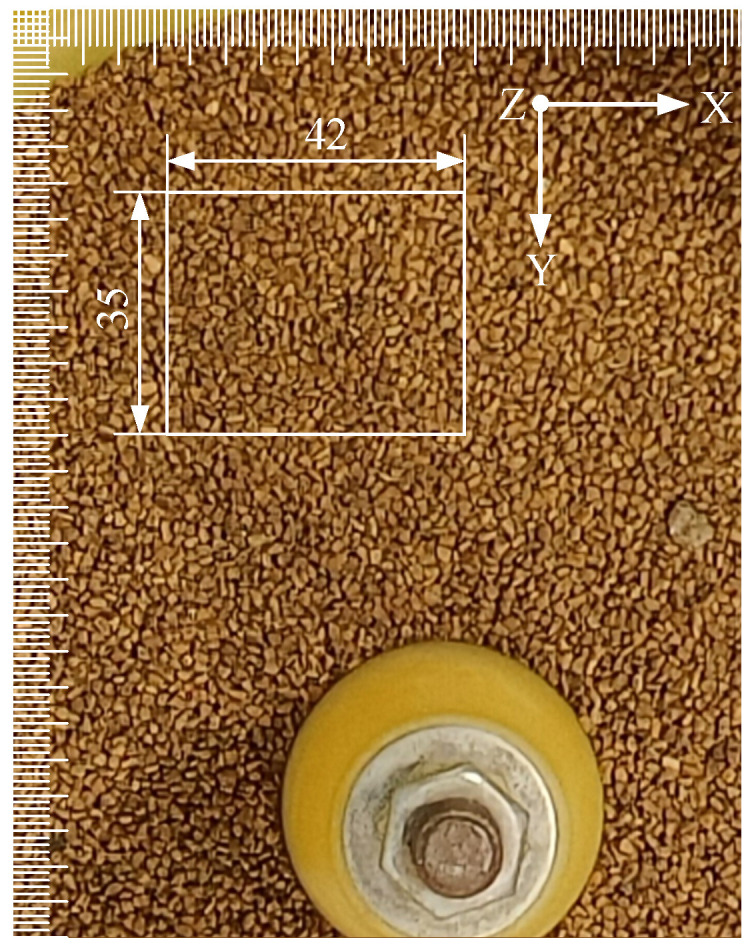
The research area.

**Figure 16:**
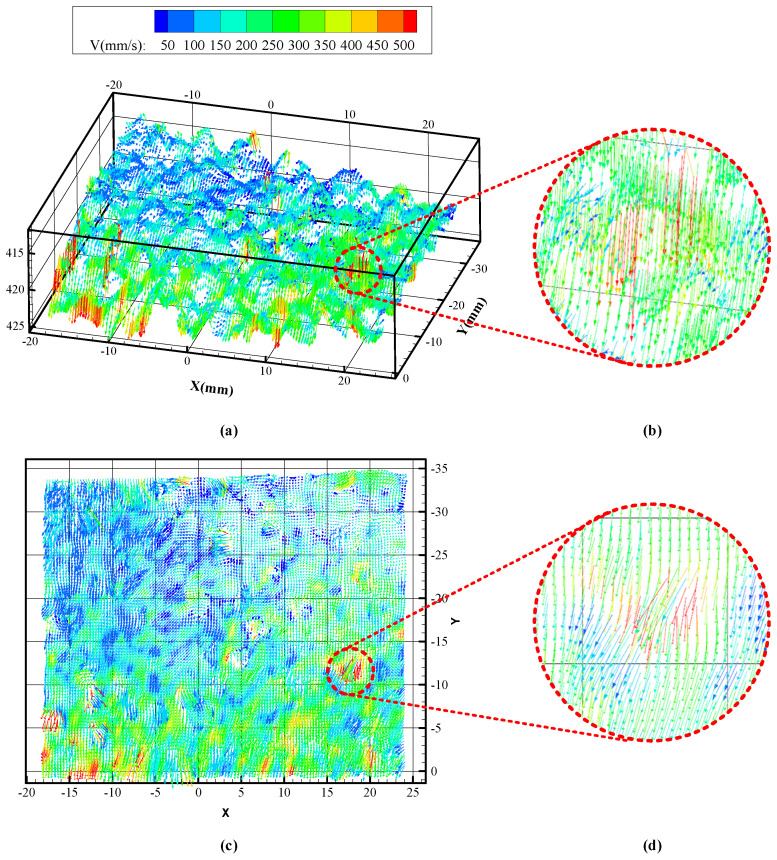
(**a**,**c**) are three-dimensional and two-dimensional schematic diagrams of the velocity field, respectively. (**b**,**d**) are the local enlarged velocity vector diagrams of the granule with the maximum velocity.

Figure 15 shows the selected research area of the granular flow field in the bowl-type vibratory finishing machine, and Figure 16a,c present the three-dimensional and two-dimensional schematic diagrams velocity field of this area.

After comparing the obtained three-dimensional velocity field of the granular media with the actual flow field motion video, it was observed that the closer the Y-axis value is to 0, the larger the velocity field value. This is because the region closer to the Y-axis of 0 is nearer to the vibration center in the bowl-type vibratory finishing machine, resulting in higher velocity. This observation aligns with the overall motion trend of the actual flow field. Upon further observation of the motion of individual granules, it was found that the granule shown in Figure 16b,d has the maximum value in the velocity field (represented by the red arrow vector). Comparison with the actual motion of the granular media revealed that this medium experienced a jump due to vibration, resulting in the highest velocity in the entire flow field. Similarly, it was observed that the regions where the maximum values of the velocity field occur are all caused by jumps of the granular media, rather than errors, indicating that the obtained velocity field perfectly matches the actual motion. Therefore, it can be concluded that the three-dimensional velocity field measurement system of the granular media is feasible in practical mass finishing processes.

## 6. Conclusions

In mass finishing, existing methods for velocity measurement in the flow field of granular media suffer from issues such as complex and expensive equipment, being limited to single-point measurements, interference with the flow field, and lack of universality across different scenarios. To address these challenges, the following measures have been undertaken:

- A single-camera-based three-dimensional velocity field measurement method of granular media with deep learning was proposed. A complete system for measuring the three-dimensional velocity field of the granular media was constructed. Displacement measurement accuracy analysis was conducted in the X and Z directions of the granular flow field by using this system. The measured displacement values exactly matched the actual applied displacement values, with mean errors in the X and Z directions of less than 0.07 mm. The measurement accuracy analysis demonstrated that the constructed measurement system accurately measures the spatial displacements of granular media in a flow field.
- The displacement measurement results of the proposed method were compared with those obtained by the traditional DIC algorithm. The measurement results of the two methods are accurate in the X direction, but the accuracy of the proposed method in the Z direction is 656.63% of that of DIC algorithm. In terms of calculation speed, the calculation speed of the proposed method is 1291.67% of that of the DIC algorithm. The performance analysis demonstrated that the proposed measurement method has significant improvement in accuracy and calculation speed compared with the traditional algorithm.
- A velocity field measurement experiment of granular media in bowl-type vibratory finishing machine was conducted. The measured three-dimensional velocity field of the media matched the actual flow field motion, validating the feasibility of the measurement system in measuring the three-dimensional flow of mass finishing flow fields.

This research not only proposed a novel method for three-dimensional reconstruction and velocity field measurement using a single-color camera, but also demonstrated a way to combine deep learning with traditional optical techniques. It is of great significance to introduce deep learning to improve traditional optical techniques and apply them to practical engineering measurements.

## Figures and Tables

**Table 1:** Camera parameters.

Device Parameters	Value	Notes
Sync Pulse Width (μs)	10	Sync Mode: Internal Sync
Resolution	1280×1024	-
Frame Rate (fps)	200	-
Exposure Time (μs)	4995	Overexposure Tip: off
Trigger Mode	-	Internal Trigger
Recording Duration (s)	5	Mode: After Trigger
Pixel Bit Depth (bit)	8	-
Stream Type	-	RAW

**Table 2:** Important parameters of the results.

Parameters	Applied Dis. (mm)										Mean
	0.5	1.0	1.5	2.0	2.5	3.0	3.5	4.0	4.5	5.0	
LiteFlowNet Measured X Dis. error (%)	1.93	−0.77	0.92	−0.05	0.56	−0.08	0.96	−0.05	2.82	−1.92	0.43
DIC Measured X Dis. error (%)	−3.94	1.55	−0.93	0.57	−0.48	0.07	−0.36	0.44	−0.27	0.78	−0.26
LiteFlowNet Measured Z Dis. error (%)	2.26	9.44	12.35	−7.89	3.31	6.04	−7.33	2.97	6.16	−7.72	1.96
DIC Measured Z Dis. error (%)	36.64	12.08	16.98	12.76	15.73	14.72	−3.02	12.40	10.13	0.30	12.87
LiteFlowNet Measured X Dis. SD	0.0039	0.0037	0.0037	0.0039	0.0036	0.0037	0.0035	0.0035	0.0032	0.0035	0.0036
DIC Measured X Dis. SD	0.0056	0.0061	0.0059	0.0054	0.0060	0.0048	0.0048	0.0043	0.0047	0.0052	0.0053
LiteFlowNet Measured Z Dis. SD	0.1140	0.1113	0.1218	0.1136	0.1035	0.1034	0.1033	0.1054	0.1071	0.1060	0.1089
DIC Measured Z Dis. SD	0.0780	0.0655	0.0644	0.0629	0.0645	0.0614	0.0619	0.0821	0.0653	0.0571	0.0663

## Data Availability

The raw data supporting the conclusions of this article will be made available by the authors on request.

## References

[1] Yang S.Q., Li W.H. (2011). Surface Finishing Theory and New Technology.

[2] Mediratta R., Ahluwalia K., Yeo S.H. (2016). State-of-the-art on vibratory finishing in the aviation industry: Industrial and academic perspective. Int. J. Adv. Manuf. Technol..

[3] Ciampini D., Papini M., Spelt J.K. (2007). Impact velocity measurement of media in a vibratory finisher. J. Mater. Process. Technol..

[4] Kumar P.P., Sathyan S. (2012). Simulation of 1D Abrasive Vibratory Finishing Process. Adv. Mater. Res..

[5] Baghbanan M.R., Yabuki A., Timsit R.S., Spelt J.K. (2003). Tribological behavior of aluminum alloys in a vibratory finishing process. Wear.

[6] Yabuki A., Baghbanan M.R., Spelt J.K. (2002). Contact forces and mechanisms in a vibratory finisher. Wear.

[7] Uhlmann E., Eulitz A., Dethlefs A. (2018). Discrete Element Modelling of Drag Finishing. Procedia Cirp.

[8] Ciampini D., Papini M., Spelt J.K. (2009). Modeling the development of Almen strip curvature in vibratory finishing. J. Mater. Process. Technol..

[9] Hashemnia K., Mohajerani A., Spelt J.K. (2013). Development of a laser displacement probe to measure particle impact velocities in vibrationally fluidized granular flows. Powder Technol..

[10] Liu J.Z., Grace J.R., Bi X.T. (2003). Novel Multifunctional Optical-Fiber Probe: I. Development and Validation. AIChE J..

[11] Hashimoto F., Johnson S.P. (2015). Modeling of vibratory finishing machines. CIRP Ann.-Manuf. Technol..

[12] Hashimoto F., Johnson S.P., Chaudhari R.G. (2016). Modeling of material removal mechanism in vibratory finishing process. CIRP Ann.-Manuf. Technol..

[13] Uhlmann E., Schmiedel C., Wendler J. (2015). CFD simulation of the Abrasive Flow Machining process. Procedia Cirp.

[14] Domblesky J., Evans R., Cariapa V. (2004). Material removal model for vibratory finishing. Int. J. Prod. Res..

[15] Grant I. (1997). Particle image velocimetry: A review. Proc. Inst. Mech. Eng. Part C J. Mech. Eng. Sci..

[16] Adrian R.J. (2005). Twenty years of particle image velocimetry. Exp. Fluids.

[17] Scarano F. (2002). Iterative image deformation methods in PIV. Meas. Sci. Technol..

[18] Hagemeier T., Börner M., Bück A., Tsotsas E. (2015). A comparative study on optical techniques for the estimation of granular flow velocities. Chem. Eng. Sci..

[19] Duan J.X., Liu X.Y., Yin Y.F. (2023). Online measurement of granular velocity of rotary drums by a fast PIV deep network FPN-FlowNet. Measurement.

[20] Horn B.K.P., Schunck B.G. (1981). Determining optical flow. Artifi. Intell..

[21] Farnebäck G. (2003). Two-frame motion estimation based on polynomial expansion. Image Analysis: 13th Scandinavian Conference, SCIA 2003, Halmstad, Sweden, 29 June–2 July 2003, Proceedings 13.

[22] Lee S.W., Paik J., Hayes M. Stereo image capture and distance estimation with an SLR digital camera. Proceedings of the IASTED International Conference on Signal and Image Processing.

[23] Dosovitskiy A., Fischer P., Ilg E., Hausser P., Hazirbas C., Golkov V., Van Der Smagt P., Cremers D., Brox T. FlowNet: Learning optical flow with convolutional networks. Proceedings of the IEEE International Conference on Computer Vision.

[24] Ilg E., Mayer N., Saikia T., Keuper M., Dosovitskiy A., Brox T. FlowNet 2.0: Evolution of optical flow estimation with deep networks. Proceedings of the 30th IEEE/CVF Conference on Computer Vision and Pattern Recognition (CVPR).

[25] Yu L.P., Pan B. (2017). Color stereo-digital image correlation method using a single 3CCD color camera. Exp. Mech..

[26] Bolles R.C., Baker H.H. (1987). Epipolar-plane image analysis: An approach to determining structure from motion. Int. J. Comput. Vis..

[27] Furukawa Y., Ponce J. (2010). Accurate, Dense, and Robust Multiview Stereopsis. IEEE Trans. Pattern Anal. Mach. Intell..

[28] Eigen D., Puhrsch C., Fergus R. Depth map prediction from a single image using a multi-scale deep network. Proceedings of the Advances in Neural Information Processing Systems 27 (NIPS 2014).

[29] Hartley R.I., Sturm P. (1997). Triangulation. Comput. Vis. Image Underst..

[30] Triggs B., McLauchlan P.F., Hartley R.I., Fitzgibbon A.W. (2000). Bundle Adjustment—A Modern Synthesis. Vision Algorithms: Theory and Practice: International Workshop on Vision Algorithms Corfu, Greece, September 21–22, 1999 Proceedings.

[31] Li X. (2005). Demosaicing by successive approximation. IEEE Trans. Image Process..

[32] Malvar H.S., He L.W., Cutler R. High-quality linear interpolation for demosaicing of Bayer-patterned color images. Proceedings of the IEEE International Conference on Acoustics, Speech, and Signal Processing.

[33] McPhail M.J., Fontaine A.A., Krane M.H., Goss L., Crafton J. (2015). Correcting for color crosstalk and chromatic aberration in multicolor particle shadow velocimetry. Meas. Sci. Technol..

[34] Yu L.P., Pan B. (2017). Full-frame, high-speed 3D shape and deformation measurements using stereo-digital image correlation and a single color high-speed camera. Opt. Lasers Eng..

[35] Hui T.W., Tang X.O., Loy C.C. LiteFlowNet: A Lightweight Convolutional Neural Network for Optical Flow Estimation. Proceedings of the 31st IEEE/CVF Conference on Computer Vision and Pattern Recognition (CVPR).

[36] Sutton M.A., Wolters W.J., Peters W.H., Ranson W.F., McNeill S.R. (1983). Determination of displacements using an improved digital correlation method. Image Vis. Comput..

[37] Dong Y.L., Pan B. (2017). A Review of Speckle Pattern Fabrication and Assessment for Digital Image Correlation. Exp. Mech..

